# Impact of diabetes mellitus on clinical outcomes of pancreatic cancer after surgical resection: A systematic review and meta-analysis

**DOI:** 10.1371/journal.pone.0171370

**Published:** 2017-02-03

**Authors:** Xinghua Lv, Wenhui Qiao, Yufang Leng, Lupeng Wu, Yanming Zhou

**Affiliations:** 1 Department of Anaesthesiology, First Hospital of Lanzhou University, Lanzhou, China; 2 Department of Hepatobiliary & Pancreatovascular Surgery, First affiliated Hospital of Xiamen University, Xiamen, China; University of Nebraska Medical Center, UNITED STATES

## Abstract

**Background and objective:**

Diabetes mellitus (DM) is a risk factor for pancreatic cancer but its impact on postoperative outcomes and long-term survival after cancer resection remains controversial. A meta-analysis of published studies was conducted to address this issue.

**Methods:**

An extensive electronic search of four databases was performed for relevant articles. Data were processed for meta-analysis using Review Manager version 5.1.

**Results:**

Seventeen observational studies involving 5407 patients were subjected to the analysis. Overall morbidity or any type of complications and mortality were comparable between diabetic and non-diabetic subjects. Overall DM has a significant negative impact on survival (risk ratio [RR], 1.24, 95% confidence interval [CI], 1.05–1.45; *P* = 0.01). Stratification by the type of DM revealed that new-onset DM (<2 years duration, RR, 1.54, 95% CI, 1.24–1.91; *P* <0.001) but not long-standing DM (≥2 years duration, RR, 1.74, 95% CI, 0.86–3.52; *P* = 0.12) was associated with reduced survival.

**Conclusions:**

Diabetes mellitus does not affect perioperative outcomes in patients undergoing surgery for pancreatic cancer. However, new-onset DM confers a negative impact on survival of pancreatic cancer in patients undergoing surgical resection.

## Introduction

Pancreatic cancer is a deadly disease, causing about 227,000 deaths worldwide every year. The exceptionally high mortality confers it as the 4th or 5th most frequent cause of cancer-related deaths in most developed countries [1]. Identification of etiological factors could enable early detection of pancreatic cancer so that it would be more amenable to treatment. One potentially important risk factor for this malignancy is diabetes mellitus (DM). A meta-analysis of 88 studies showed a strong association between DM and pancreatic cancer development (pooled odds ratio (OR), 1.97, 95% confidence interval [CI], 1.78–2.18) [2]. Hyperinsulinemia and insulin-resistance have been proposed as potential biologic mechanisms

Pancreatectomy is the only treatment that can offer long-term survival in patients with pancreatic cancer at present. Some publications have reported that DM is associated with an increased risk of postoperative complications or worse survival outcomes following resection of pancreatic cancer [3–5], but others have failed to demonstrate such an association [6–8]. In the light of this controversy, we made a meta-analysis of published studies to address this issue.

## Materials and methods

### Study selection

This study followed the Preferred Reporting Items for Systematic Reviews and Meta-Analyses (PRISMA) guideline [9]. An electronic search of the literature was conducted in PubMed, Web of Science, Cochrane Library, and China National Knowledge Infrastructure from the time of inception to June 2016, using the following terms: “pancreatic cancer”, “diabetes mellitus”, “pancreatic resection”, “pancreaticoduodenectomy”, “post-operative outcomes”, and “prognosis”. Manual search of reference lists of all retrieved articles was carried out to identify additional studies.

### Criteria for inclusion and exclusion

Published studies in the English or Chinese language comparing outcomes in DM and non-DM patients undergoing surgical resection with curative intent for pancreatic cancer were included. Letters, reviews, abstracts, editorials, expert opinions, non-English language papers, animal or in vitro studies, studies lacking control groups, studies with a small sample size (<10 in number), studies evaluating treatment in patients with unresectable diseases, and studies that contained patients with other periampullary adenocarcinomas (duodenal, ampullary, and biliary) without separate assessments were excluded.

### Data extraction and outcomes of interest

Two reviewers (XL and YZ, respectively) independently extracted relevant data regarding the characteristics of study and outcomes of interest from each selected article by using standardized data extraction forms. Discrepancies were resolved by discussion until consensus was achieved.

The outcomes of interest analyzed included (a) clinicopathologic characteristics; (b) postoperative morbidity and mortality; and (c) overall survival (OS).

### Assessment of methodological quality

The methodological quality of the included studies was assessed by using the Newcastle-Ottawa Scale. Scores were assigned for patient selection, comparability of the study groups, and outcome assessment [10].

### Statistical methods

The effect measures estimated were OR with a 95% CI for dichotomous variables and weighted mean difference (WMD) with a 95% CI for continuous data. The relative risk ratio (RR) with 95% CI was used to assess the prognostic value of DM, where an observed RR >1 implied a worse survival for DM group. To do this, the hazard ratio (HR) was directly considered as RR. To assess heterogeneity across studies, the I^2^ statistic was calculated and a value >50% was interpreted as statistically significant. A funnel plot based on the survival outcome was conducted to explore the possibility of publication bias. Statistical analyses were performed with Review Manager version 5.1 (The Cochrane Collaboration, Software Update, Oxford). A value of *P* < 0.05 was considered statistically significant.

## Results

### Selection of studies

**Fig 1:**
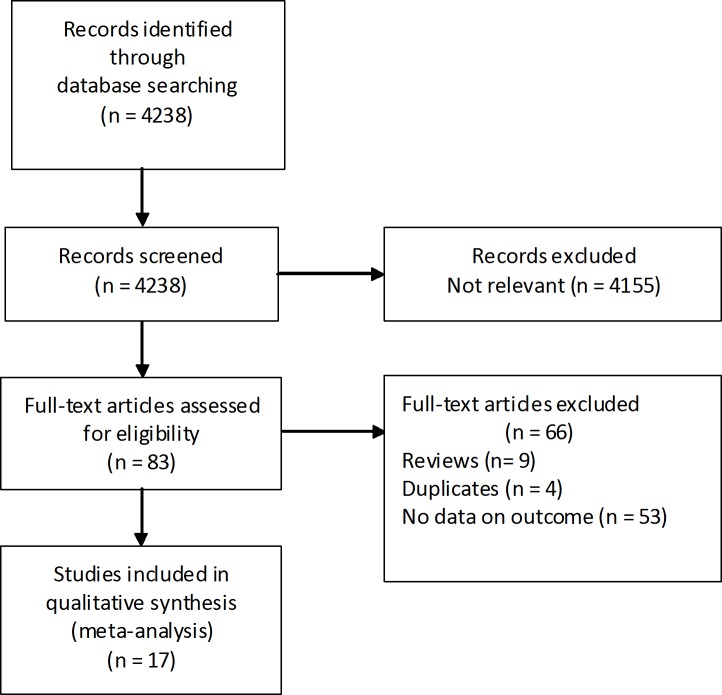
Flow diagram of included studies.

Fig 1 presents the flowchart of selection for study. Among 4238 references identified by the initial search, 17 [3–8, 11–21] were finally met the inclusion criteria and suitable for analysis. The main characteristics of these 17 studies are summarized in Table 1. Most reports were conducted in the United States (n = 7) and Asia (n = 7), followed by Europe (n = 3). Of the two studies conducted at the same institution [4,5], the former mainly assessed the impact of DM on long-term survival, and the latter mainly assessed the impact of DM on perioperative morbidity and mortality. All identified studies were observational studies involving a total of 5407 patients, including 1669 in DM group and 3738 in non-DM group. The sample size of these studies varied from 83 to 1071 patients. The percentage of DM ranged from 8.8% to 56.3%. Details regarding DM definition were described in 13 articles [4,5,7,8,13–21]. Operation types were presented in 13 articles covering 4108 patients [3–5,7,8,11,12,16–21]. In total, 3333 (81.3%) patients underwent pancreaticoduodenectomy, 576 (14.0%) patients underwent left pancreatectomy, and 199 (4.7%) patients underwent total pancreatectomy.

**Table 1:** Clinical background of studies included in the meta-analysis.

Reference	Year	Country	No	DM	Definition	TR	Morbidity	Mortality	Survival	NOS
				n (%)	of DM	PD/DP/TP	n (%)	n (%)		
Sperti [3]	1996	Italy	113	62 (54.8)	NA	77/23/13	45 (39.8)	17 (15)	12%^a^	6
Chu [4]	2010	USA	209	93 (45)	Diabetic history or FPG≥126 mg/dl or	183/24/2	NA	4 (2)	16^b^	6
					RBG ≥200 mg/dl on 2 separate days					
Chu [5]	2010	USA	251	116 (46.2)	Diabetic history or FPG≥126 mg/dl or	220/29/2	196 (78.1)	9 (3.6)	NA	6
					RBG ≥200 mg/dl on 2 separate days.					
Olson [6]	2010	USA	160	14 (8.8)	NA	NA	NA	NA	NA	6
Dandona [7]	2011	USA	355	116 (32.7)	Diabetic history	355/0/0	NA	NA	NA	6
Hartwig [11]	2011	Germany	1071	151 (14.1)	NA	712/199/160	NA	24 (2.2)	NA	6
Barbas [12]	2012	USA	203	51 (25.1)	NA	203/0/0	148 (72.9)	7 (3.4)	NA	6
Ben [13]	2012	China	396	107 (27.0)	Diabetic history or FBG ≥126 mg/dL	NA	NA	NA	4%^a^	7
					or PBG ≥200 mg/dl					
Sahin [14]	2012	USA	544	144 (26.5)	Diabetic history	NA	NA	NA	31.3^b^	7
He [15]	2013	China	199	90 (44.7)	Diabetic history or FPG≥126 mg/dl or	NA	38 (19.1)	NA	16%^a^	7
					RBG ≥200 mg/dl on 2 separate days.					
Zheng [16]	2013	China	302	113 (37.4)	FPG≥126 mg/dl or RBG ≥200 mg/dl on	302/0/0	NA	10 (3.3)	NA	6
					2 separate days					
Malleo [8]	2013	Italy	602	120 (19.9)	Diabetic history or FPG≥126 mg/dl or	494/108/0	252 (41.9)	3 (0.5)	NA	6
					RBG ≥200 mg/dl on 2 separate days					
Hart [17]	2014	USA	488	275 (56.3)	Diabetic history or FBG ≥126 mg/dL	382/84/22	NA	NA	24.4^b^	6
Xu [18]	2015	China	83	28 (33.7)	FPG≥126 mg/dl or RBG ≥200 mg/dl on	62/21/0	NA	NA	24.8^c^	7
					2 separate days					
Duan [19]	2016	China	179	81 (45.3)	Diabetic history or FPG≥126 mg/dl or	179/0/0	NA	5 (2.8)	NA	6
					RBG ≥200 mg/dl on 2 separate days					
Lee [20]	2016	Korea	147	77 (52.4)	Diabetic history or RBG ≥200 mg/dl	96/51/0	81 (55.1)	0	31.5%^c^	7
Zhu [21]	2016	China	105	31 (29.5)	FPG≥126 mg/dl or RBG ≥200 mg/dl on	68/37/0	NA	NA	20%^c^	7
					2 separate days					

DM, diabetes mellitus; TR, type of resection; PD, pancreaticoduodenectomy; DP, distal pancreatectomy; TP, total pancreatectomy; RBG, random blood glucose; NA, not available; FBG, fasting blood glucose; PBG, postprandial blood glucose; NOS, Newcastle–Ottawa Scale.

^a^ 5-years overall survival

^b^ Median overall survival (months)

^c^ 3-year overall survival.

### Meta-analysis

Table 2 shows the results for the outcomes.

**Table 2:** Meta-analysis of short and long-term outcomes.

Outcome of interest	No. of studies	No.of patients	OR/WMD	95% CI	*P*-value	I^2^ (%)
Characteristics of patients						
Gender	10	DM = 1071, Non-DM = 1763	0.81	0.69, 0.95	0.01	0
Age	7	DM = 764, Non-DM = 1246	1.66	-0.66,3.17	0.20	76
Body mass index	3	DM = 472, Non-DM = 446	1.45	0.60, 2.30	<0.001	56
Smoking history	3	DM = 511, Non-DM = 830	0.97	0.75, 1.25	0.80	24
Jaundice	5	DM = 535, Non-DM = 1239	0.97	0.70, 1.34	0.85	52
Type of operation	6	DM = 647, Non-DM = 1029	1.15	0.89, 1.49	0.28	0
Duration of surgery (min)	7	DM = 566, Non-DM = 1103	-4.38	-16.38, 7.62	0.47	0
Blood transfusion	3	DM = 313, Non-DM = 687	1.12	0.81, 1.56	0.50	0
Tumor site	6	DM = 645, Non-DM = 921	1.21	0.80, 1.81	0.37	58
Tumor size	8	DM = 744, Non-DM = 1217	0.27	0.12, 0.42	<0.001	14
Node involvement	4	DM = 448, Non-DM = 940	1.09	0.85, 1.38	0.50	12
Poor differentiation	7	DM = 920, Non-DM = 1705	1.22	1.02, 1.47	0.03	49
Perineural invasion	6	DM = 645, Non-DM = 1492	1.27	0.85, 1.90	0.24	57
Lymphovascular invasion	6	DM = 645, Non-DM = 1492	1.18	0.96, 1.45	0.12	0
Stage	4	DM = 592, Non-DM = 773	0.75	0.49, 1.15	0.18	10
Hard pancreatic texture	4	DM = 430, Non-DM = 904	3.48	2.34, 5.18	<0.001	56
Positive margin	5	DM = 732, Non-DM = 1300	1.18	0.93, 1.48	0.17	0
Postoperative outcomes						
Overall morbidity	4	DM = 403, Non-DM = 796	0.90	0.59, 1.39	0.65	54
Pancreatic fistula	6	DM = 453, Non-DM = 914	0.88	0.50, 1.54	0.65	65
ISGPF B +C fistula	2	DM = 236, Non-DM = 617	0.69	0.20, 2.44	0.57	65
Delayed gastric emptying	6	DM = 453, Non-DM = 914	1.08	0.75, 1.55	0.69	0
Abdominal collection or abscess	3	DM = 313, Non-DM = 687	0.84	0.53, 1.35	0.48	0
Biliary fistula	2	DM = 197, Non-DM = 552	0.48	0.14, 1.61	0.24	50
Wound infection	2	DM = 193, Non-DM = 205	1.11	0.67, 1.85	0.68	0
Cardiac complications	3	DM = 317, Non-DM = 718	1.53	0.83, 2.80	0.17	31
Respiratory complications	4	DM = 394, Non-DM = 785	0.89	0.54, 1.46	0.64	0
Renal dysfunction	3	DM = 317, Non-DM = 715	1.75	0.96, 3.18	0.07	17
Mortality	3	DM = 317, Non-DM = 715	1.60	0.58, 4.42	0.36	0
Overall survival						
Overall DM	11	DM = 1103, Non-DM = 2635	1.24^a^	1.05, 1.45	0.01	64
Long-standing DM	3	DM = 79, Non-DM = 540	1.74^a^	0.86, 3.52	0.12	79
New-onset DM	3	DM = 208, Non-DM = 540	1.54^a^	1.24, 1.91	<0.001	0

OR, odds ratio; WMD, weighted mean difference; CI, confidence interval; ISGPF, International Study Group of pancreatic fistula; DM, diabetes mellitus

^a^ risk ratio.

Compared with non-DM patients, DM patients had higher prevalence of male sex (OR, 0.81, 95% CI, 0.69–0.95; *P* = 0.01) and greater body mass index (WMD, 1.45, 95% CI, 0.60–2.30; *P* <0.001). There was no significant difference in age, smoking history and the presence of jaundice between the two groups. The operative variables including operation type, duration of surgery and transfusion were comparable between the two groups. Pathologically, DM patients had significantly higher prevalence of poor differentiation (OR, 1.22, 95% CI, 1.02–1.47; *P* = 0.03) and hard pancreatic texture (OR, 3.48, 95% CI, 2.34–5.18; *P* <0.001) and were more likely to have larger tumor sizes (WMD, 0.27, 95% CI, 0.12–0.42; *P* <0.001). There was no significant difference in tumor location, lymph node involvement, perineural invasion, lymphovascular invasion, cancer stage, and the margin status between the two groups.

**Fig 2:**
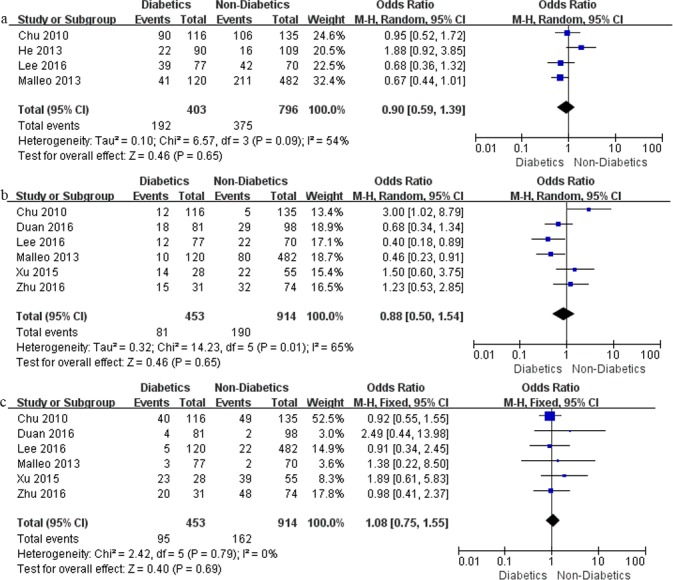
Meta-analysis on postoperative outcomes: (a) overall morbidity; (b) pancreatic fistula; and (c) delayed gastric emptying.

On postoperative outcomes analysis, diabetics were not at increased risk for development of overall morbidity (OR, 0.90, 95% CI, 0.59–1.39; *P* = 0.65), pancreatic fistula (OR, 0.88, 95% CI, 0.50–1.54; *P* = 0.65), delayed gastric emptying (OR, 1.08, 95% CI, 0.75–1.55; *P* = 0.69) (Fig 2), as well as other complications and mortality.

**Fig 3:**
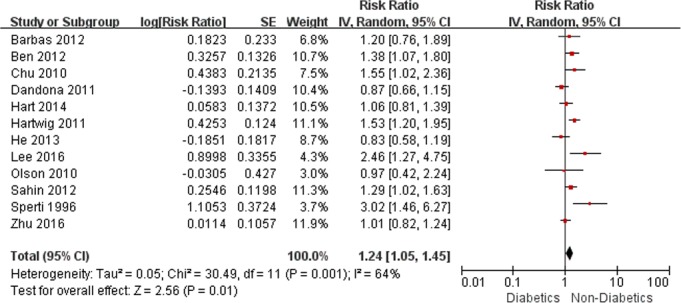
Meta-analysis on overall survival.

The result of analysis on survival showed that DM had a significant negative impact on prognosis (RR, 1.24, 95% CI, 1.05–1.45; *P* = 0.01) (Fig 3). Subsequent analyses were restricted to nine studies [3.5, 11–17,20] that reported multivariate-adjusted estimates yielded similar results (RR, 1.35, 95% CI, 1.13–1.62; *P* = 0.001). Stratification by the type of DM revealed that new-onset DM (<2 years duration, RR, 1.54, 95% CI, 1.24–1.91; *P* <0.001) but not long-standing DM (≥2 years duration, RR, 1.74, 95% CI, 0.86–3.52; *P* = 0.12) was associated with reduced survival.

### Publication bias

**Fig 4:**
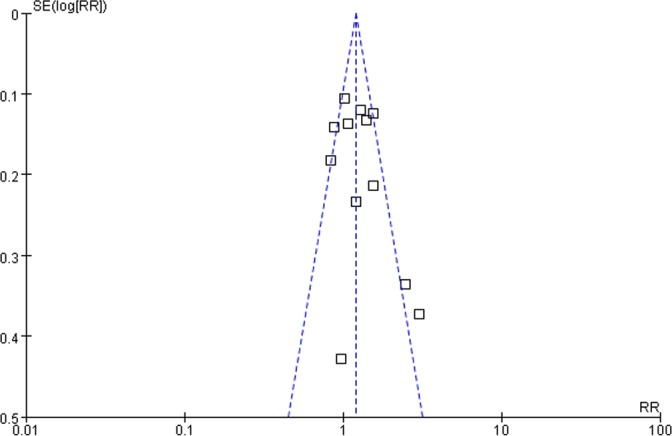
Funnel plot for the results from overall survival.

A funnel plot demonstrated that two of the studies fell outside the limits of the 95% CI for the impact of overall DM on survival, suggesting the presence of publication bias (Fig 4).

## Discussion

Diabetes mellitus is reported to be associated with increased events of cardiovascular and renal dysfunction and have an adverse effect on postoperative outcomes of vascular, hepatic and gastric surgeries [22–24]. In the field of pancreatic surgery, Srivastava et al. [25] found that DM was a risk factor associated with an increased incidence of pancreatic fistula in their 120 patients with pancreatic and periampullary tumors who underwent pancreaticoduodenectomy (OR, 4.60, 95% CI, 1.23–17.18). On the other hand, DeOliveira et al. [26] reported that DM was not a significant indicator for increased occurrence of overall complications or any type of complications in their 633 patients with various benign and malignant diseases after pancreaticoduodenectomy. These conflicting results might be partially explained by the heterogeneous patient groups studied. By limiting analysis to patients undergoing resection for pancreatic cancer, a condition with a low risk of pancreatic fistula compared with other histologic diagnoses [27], the current study shows that the incidence of overall postoperative morbidity and cardiovascular and renal complications are comparable in diabetic and nondiabetic patients. This may partially reflect careful patient selection for operation and current perioperative management for patients at high risk.

Pancreatic fistula is the principal complication related to pancreatic surgery and may cause fatal consequences. A soft pancreatic texture and a small pancreatic duct (<3 mm) are well recognized as risk factors predisposing the development of pancreatic fistula [28]. Our analysis showed that diabetic patients usually had a low frequency of soft pancreatic texture as compared with non-diabetic patients. Furthermore, the frequency of a small pancreatic duct or diameter of the pancreatic duct was found to be similar between diabetic and non-diabetic patients [5,8]. Not surprisingly, we failed to demonstrate any difference in the rate and severity of pancreatic fistula between the two groups.

The prognostic value of DM in pancreatic cancer is disputable. Some investigators identified no significant survival difference between diabetic and nondiabetic patients [29,30], while others noted that survival was reduced in DM cohorts compared with nondiabetic group [31,32]. However, most patients in these studies suffered from unresectable tumors, nor was subset analysis for survival carried out on the basis of surgical cohorts. A previous published meta-analysis restricted to patients with resectable tumors included 8 studies and found that DM was associated with a worse OS after curative resection of pancreatic cancer (HR, 1.32, 95% CI, 1.46–1.60) [33]. Similarly, the detrimental effect of DM on prognosis is also demonstrated in the present update. Our strengths lie within the addition of more recent four published studies [15,17,20,21] and therefore increase the power of the estimates, providing further validation for these findings. The subpopulation of DM patients was characterized by more prevalence of comorbidities and larger tumor size, and hence may have contributed to a confounding effect. However, restricting the analysis to studies that reported multivariate-adjusted estimates did not alter the overall meta-analysis results, meaning that DM itself is an unfavorable prognostic factor rather than a confounder.

Stratification according to the duration of DM showed that new-onset DM is significantly associated with reduced survival, while long-standing DM does not affect OS significantly. An epidemiological study [2] that examined the relationship between DM and pancreatic cancer also showed a strong association between new-onset DM and survival of pancreatic cancer patients, as compared with long-standing DM. There is evidence that new-onset DM in pancreatic cancer is likely a paraneoplastic phenomenon mediated by tumor-secreted products [34]. It is plausible that the same mechanism by which DM may cause pancreatic cancer may also accelerate pancreatic cancer progression and affect survival.

Owing to the positive link of DM with pancreatic cancer, anti-diabetic drugs may play a role in the prevention and treatment of pancreatic cancer. Experimental evidence has demonstrated that metformin, a common antidiabetic drug in the treatment of DM2, can inhibit the growth of pancreatic cancer cells via a mechanism related to its effect on disrupting crosstalk between insulin/ insulin-like growth factor 1 (IGF1) and G protein-coupled receptor (GPCR) signaling pathways, a system implicated in autocrine-paracrine stimulation of a variety of malignancies, including pancreatic cancer [35]. A recent study of 171 pancreatic cancer patients who underwent surgical resection showed that metformin use was associated with better overall survival (*P* = 0.035) [36]. But as the study was limited to its retrospective design, future prospective research would be potentially meaningful.

The main limitation of this meta-analysis is that all included studies were observational studies that related to recall and information bias. Patient characteristics, operative procedures, perioperative care, and follow-up protocols varied widely between the included studies. These factors may affect heterogeneity of the outcomes. In addition, the prognostic significance of new-onset DM is likely to be underestimated due to the small number of patients. Further studies with larger patient samples are warranted.

## Conclusions

In conclusion, DM does not seem to affect perioperative outcomes in patients undergoing surgery for pancreatic cancer. However, new-onset DM confers a negative impact on survival of pancreatic cancer in patients undergoing surgical resection.

## Supporting information

S1 File PRISMA 2009 checklist. (DOCM)
